# Optimization of Sonotrode Ultrasonic-Assisted Extraction of Proanthocyanidins from Brewers’ Spent Grains

**DOI:** 10.3390/antiox8080282

**Published:** 2019-08-06

**Authors:** Beatriz Martín-García, Federica Pasini, Vito Verardo, Elixabet Díaz-de-Cerio, Urszula Tylewicz, Ana María Gómez-Caravaca, Maria Fiorenza Caboni

**Affiliations:** 1 Department of Analytical Chemistry, Faculty of Sciences, University of Granada, Avd. Fuentenueva s/n, 18071 Granada, Spain; 2 Department of Agricultural and Food Sciences, University of Bologna, Piazza Goidanich 60, (FC) 47521 Cesena, Italy; 3 Department of Nutrition and Food Science, University of Granada, Campus of Cartuja, 18071 Granada, Spain; 4 Institute of Nutrition and Food Technology ‘José Mataix’, Biomedical Research Center, University of Granada, Avda del Conocimiento sn, 18100 Armilla, Granada, Spain; 5 Interdepartmental Centre for Agri-Food Industrial Research, Alma Mater Studiorum, Università di Bologna, via Quinto Bucci 336, 47521 Cesena (FC), Italy

**Keywords:** Box–Behnken design, proanthocyanidins, Brewers’ spent grains, sonotrode ultrasonic-assisted extraction, HPLC-fluorometric detector (FLD)–MS

## Abstract

Brewing spent grains (BSGs) are the main by-product from breweries and they are rich of proanthocyanidins, among other phenolic compounds. However, literature on these compounds in BSGs is scarce. Thus, this research focuses on the establishment of ultrasound-assisted extraction of proanthocyanidin compounds in brewing spent grains using a sonotrode. To set the sonotrode extraction up, response surface methodology (RSM) was used to study the effects of three factors, namely, solvent composition, time of extraction, and ultrasound power. Qualitative and quantitative analyses of proanthocyanidin compounds were performed using HPLC coupled to fluorometric and mass spectrometer detectors. The highest content of proanthocyanidins was obtained using 80/20 acetone/water (*v*/*v*), 55 min, and 400 W. The established method allows the extraction of 1.01 mg/g dry weight (d.w.) of pronthocyanidins from BSGs; this value is more than two times higher than conventional extraction.

## 1. Introduction

Barley is the basic raw material for brewing. Phenolic compounds identified in barley include flavonoids, phenolic acids, and proanthocyanidins (PCs) [1,2]. There are more than 50 PCs in barley, comprising flavan-3-ol oligomers and their polymers [3]. The oligomers include dimers (prodelphinidin B3 and procyanidin B3), trimers, tetramers, and pentamers, while polymers are formed by oxidation and polymerization of simple flavan-3-ols [4]. Barley PCs ranged from 25 to 250 mg/100 g of grain [5,6,7,8]. Among them, proanthocyanidin trimers, such as catechin–gallocatechin–catechin (C–GC–C), prodelphinidin B3 and procyanidin B2 [9] are the most representative in barley. In addition, hops also contribute to the proanthocyanidin content in brewing spent grains (BSGs); in fact, according to several authors, this ingredient contains high amounts of catechin and procyanidins [10,11]. 

Furthermore, PCs showed anti-bacterial [12], anti-viral [13], anti-carcinogenic [14], anti-inflammatory [15], and cardioprotective effects [16]. Some studies demonstrated the potential of PCs for prevention or treatment of oxidative stress-associated diseases due to their antioxidant capacity [17]. In addition, PCs are easily extracted, affordable, and demonstrated low toxicity [17]. 

During the process of brewing, many BSGs are generated from barley grains after separation of the wort, and they consist of the residues from malted barley which could contain adjuncts (non-malt sources of fermentable sugars) such as wheat, rice, or maize and hop added during mashing [1]. Consequently, this by-product is rich in protein, fibers, arabinoxylans, and β-glucan, and also contains PCs in low concentration; thus, its reutilization could be useful for the food industry, and offers an opportunity for cereal-based baked and extruded products with acceptable sensory and nutritional characteristics [1]. 

In this sense, the challenge is to increase the efficient collection of PC-rich extracts with high bioactivity by the optimization of the extraction process. Thus far, conventional solid/liquid extraction was often used, employing as an extraction solvent a mixture of acetone and water in proportions from 50/50 to 80/20 [4,8,18,19] due to the large number of OH groups in PCs. In addition, bath-ultrasound-assisted extraction is the most used extraction technique. Some authors carried out pressurized solvent extraction, which is a static solid/liquid extraction with high pressure and eventually high temperature in stainless-steel extraction cells. Nevertheless, conventional extractions using ultrasonic-assisted extraction seem to be the best choice, since it is an economical technique, can be performed at atmospheric pressure and ambient temperature, and it could be developed on an ultrasound (US) bath or even with an US probe (or sonotrode) [20,21].

To carry out the determination of PCs in cereal, high-performance liquid chromatography (HPLC) is the analytical technique usually applied to this aim. In many instances, this technique was coupled to a diode array detector (DAD), fluorometric detector (FLD), and mass spectrometer detector (MSD) [8,22,23], or matrix-assisted laser desorption/ionization time-of-flight (MALDI-TOF) analysis [24].

In view of the above, the objective of this work was to evaluate the recovery of proanthocyanidins from BSGs by establishing a sonotrode ultrasonic-assisted extraction method. For that purpose, response surface methodology (RSM) was performed to evaluate extraction parameters with an experimental Box–Behnken design.

## 2. Materials and Methods 

### 2.1. Samples

Brewers’ spent grain (BSG) samples were obtained in a micro-brewing plant after pilsner beer production (Mastrobirraio, Cesena, Italy, 44°08′00″ north (N), 12°14′00″ east €).

### 2.2. Chemicals

HPLC-grade water and solvents were purchased from Merck KGaA (Darmstadt, Germany). Catechin was purchased from Sigma-Aldrich (St. Louis, MO).

### 2.3. Experimental Design

Response surface methodology (RSM) is the most popular tool for modeling. In RSM, statistical models and polynomial equations are always combined to provide an approximate relationship between the dependent and independent variables [25]. In the present work, a Box–Behnken design (BBD) with three factors was carried out in order to optimize the extraction parameters of proanthocyanidins in BSGs. The parameters of ultrasound-assisted extraction (US) can be divided into US parameters (ultrasound frequency, duration, acoustic power/intensity, and treatment mode) and non-US parameters (solvent type, solvent/sample ratio, particle size, temperature) [25]. In this work, the factors investigated were acetone/water (X1), time (X2), and potency (X3), with three levels for each factor, and the response variable (Y) was the sum of the total content of proanthocyanidins (PCs). The range for the percentage of acetone/water was chosen based on the conditions previously established in other works (50, 75, and 100%) [4,8]; the extraction time (5, 30, and 55 min) and the US power (80, 240, and 400 W) were the same as those previously used in a study where a sonotrode US was employed to optimize these parameters for the extraction of phenolic compounds from *Psidium guajava* L. leaves [26]. The design consisted of 15 combinations including three center points (Table 1), and the experiments were randomized to maximize the effects of unexplained variability in the observed response, due to extraneous factors.

The determination of optimal US sonotrode parameters was carried out using STATISTICA 7.0 (2002, StatSoft, Tulsa, OK).

### 2.4. Extraction of Proanthocyanidins from Brewers’ Spent Grains by Sonotrode Ultrasonic Extraction

The extraction was achieved with a US sonotrode UP400St (Hielscher Ultrasonics GmbH, Teltow, Germany) and, during the extraction, an ice bath was used to avoid rises in temperature. The temperature ranged between 23 and 25 °C in all extractions, and it was measured with a thermometer at the end of each extraction. The percentage of acetone/water, the extraction time, and the US power were varied according to the experimental design. After the extraction, samples were centrifuged at 1000× g for 10 min; supernatants were collected, evaporated, and reconstituted in 1 mL of methanol/water (1/1, *v*/*v*). The final extracts were filtered through 0.2-μm polytetrafluoroethylene (PTFE) syringe filters and stored at −18 °C until the analyses.

### 2.5. Conventional Extraction of Proanthocyanidins

The results obtained by the US sonotrode at the optimal conditions were compared with a PC extract from BSGs obtained via conventional solid/liquid extraction. The extraction methodology was carried out according to Carciochi et al. [27]. Briefly, BSGs were subjected to mechanical agitation with a *w*/*v* ratio of 1/30, temperature of 80 °C, 72/28 ethanol/water (*v*/*v*), and an extraction time of 60 min.

### 2.6. Determination of Proanthocyanidins in Brewing Spent Grain Extracts by HPLC-FLD-MS Analysis

The separation of proanthocyanidins was performed on an Agilent 1200 Series HPLC system (Agilent Technologies, Santa Clara, CA) equipped with a binary pump delivery system, a degasser, an autosampler, and FLD and MS detectors (MSD, model G1946A, Santa Clara, CA, USA). A Develosil Diol 100 Å column (250 × 4.6 mm, 5 µm particle size) purchased from Phenomenex (Torrance, CA, USA) was used for the analyses.

All solvents were HPLC-grade and were filtered in a filter disc of 0.45 μm. According to Robbins et al. [28], the elution binary gradient consisted of CH3CN/HOAc, 98/2 (*v/v)* as solvent A, and CH_3_OH/H_2_O/HOAc 95/3/2 *v/v/v* as solvent B. The analyses started with 7% of phase B from 0 to 3 min. Thus, solvent B was increased to 37.6% (from 3.1 to 57 min) and then to 100% B over the next 3 min for 7 min. After that, the initial condition was established, and they were maintained for 16 min. The injection volume was 5 μL and all the analyses were run at 35°C. Additionally, fluorescence detection was conducted with an excitation wavelength of 230 nm and an emission wavelength of 321 nm.

Moreover, identification of proanthocyanidins was carried out by HPLC-MS according to Verardo et al. [8]. Furthermore, quantification of PCs was done employing a calibration curve of (+)-catechin done from the limit of quantitation (LOQ) to 250 μg/mL (LOQ = 0.193 µg/mL). In addition, the quantification of dimers, trimers, tetramers, pentamers, and the polymers was done using the correction factors suggested by Robbins et al. [28].

## 3. Results and Discussion

### 3.1. Determination of Proanthocyanidin Compounds in Brewers’ Spent Grains

Table 1 shows the sum of the total content of proanthocyanidins according to the experimental design (Table 1).

A total of 11 PCs were identified in BSGs according to their degree of polymerization and their mass spectra. As shown in Table 2 (and in Figure S1), the elution order depended on the number of flavan-3-ol units. Therefore, monomers eluted first and then the different oligomers eluted. In addition, for the same degree of polymerization, a higher degree of galloylation meant a higher retention time [8]. 

Moreover, quantification of PCs in brewing by-products was carried out using HPLC-FLD. The calibration curve of catechin was used to quantify the PCs. The correction factors were applied according to Robbins et al. [28]. The concentration values of PCs obtained in each experiment in the BBD are presented in Table 3. Briefly, the total content of PCs varied from 540.04 µg∙g^−1^ dry weight (d.w.) to 1002.31 µg∙g^−1^ d.w. Comparing the quantification of each compound, experiment 11, whose parameters of extraction were 75% acetone, 5 min, and 400 W of US power, recovered higher amounts of catechin/epicatechin, dimers, trimers, and tetramers than the rest of the experiments. Finally, the major concentrations of procyanidin pentamer, the polymer, and the total content of PCs were obtained in experiment 12 with 75% acetone, 55 min, and 400 W of US power. 

Proanthocyanidins were grouped as monomer, dimers, trimers, tetramers, pentamers, and polymers.

### 3.2. Fitting the Model

The regression model for the BBD was fitted employing the data from Table 1 in order to find the combined effect of extraction time, acetone/water ratio, and sonotrode US power on the response variable during the sonotrode US. For that, an analysis of variance (ANOVA) with 95% confidence level was employed to analyze the regression model and to evaluate the effect of the coefficients for each factor (linear and quadratic terms) and the interaction between them (cross-product term). In fact, the evaluation of the model was carried out according to the significance of the regression coefficients which are displayed in Table 4. According to other works, the level of significance could be fixed at α < 0.1 in order to increase the number of significant terms [26]. In the present work, the model was analyzed at α < 0.05 and α < 0.1 The significant variables for the total content of PCs were the intercept (X_0_) (*p =* 0.000426), the linear effect of acetone/water (X_1_) (*p* = 0.058033) and its quadratic effect (X_11_) (*p* = 0.018319), the linear effect of time (X_2_) (*p* = 0.060966), and the quadratic effect of the power (X_33_) (*p* = 0.085914). Furthermore, ANOVA revealed that the model presented a high correlation between the factors and the response variables with a coefficient of determination (*R^2^*) of 0.8999 (Table 4). In addition, the *p*-value of the regression model and the *p*-value of the lack-of-fit (LOF) were also used to verify the adequacy of the model. In fact, a high correlation term, a significant regression model (*p* < 0.05), and a non-significant LOF (*p* > 0.05) demonstrated the validity of the model (Table 4).

#### 3.2.1. Analysis of Response Surfaces

**Figure 1:**
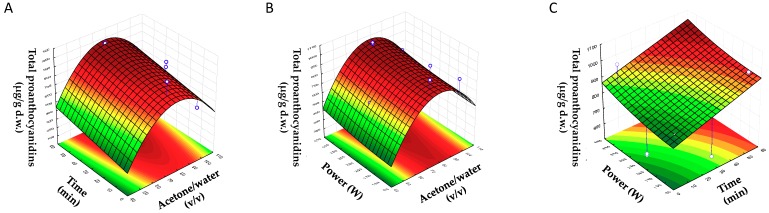
Response surface plots showing the combined effects of process variables for total proanthocyanidins: (**A**) acetone/water (% (*v/v*)) vs. time (min); (**B**) acetone/water (% (*v/v*)) vs. ultrasound (US) power (W); (**C**) time (min) vs. US power (W).

In order to determine the optimal value of each factor for the extraction of PCs from BSGs, response surfaces were plotted. Each pair of variables was depicted in three-dimensional surface plots, while the other factor was kept constant at a central level. Figure 1 shows the three-dimensional plots for the effects of acetone/water (% (*v/v*)) (X1) with time (X2), acetone/water (% (*v/v*)) (X1) with US power (X3), and time (X2) with US power (X3) on the concentration of the total content of PCs.

In Figure 1A,B, it can be observed that the response of the total content of PCs increased when the concentration of acetone increased at first. After that, a decrease in response was observed when the maximum response was achieved. This shape was a consequence of the quadratic effect of acetone, which had a negative value, showing that an increase in this parameter more than a certain value tended to decrease the response. For example, Figure 1A shows an increase in total concentration of PCs if the content of acetone rose until the maximum value (75–85%), for which the increase time caused a slight increase in the total concentration of PCs. Additionally, in Figure 1B, an increase in the content of total PCs up to 70–85% acetone was observed where it started to reduce, whereas the response increased slightly at 70–85% if the power increased. At last, Figure 1C shows the positive linear effect of time and power on the response; there was an increase in response with time and power.

#### 3.2.2. Optimization of Sonotrode US Parameters

The optimal conditions were selected through the three-dimensional (3D) plots to obtain the highest content of PCs from BSGs, as shown in Table 5.

Briefly, optimal extraction conditions were 80% acetone/water (*v*/*v*), 55 min, and 400 W for US power. The final step of the RSM after selecting the optimal conditions was to verify the accuracy of the mathematical model. For that, an extraction at optimal conditions was done with the same methodology; the obtained value did not report significant differences with the predicted value. 

According to the results, the maximum content of PCs was obtained at 80% acetone/water, because PCs with a high degree of polymerization were the most concentrated, and they were better extracted at a high percentage of acetone, since they were less polar than the other PCs, increasing their solubility in this solvent. Also, acetone was not an efficient solvent when used pure, showing good results when it was combined with water. This occurred due to increased solvation provided by the presence of water. Additionally, at a high time of extraction and maximum power, cell walls were disrupted, releasing proanthocyanidins from the cell constituents. The predicted values of the model were in accordance with the experimental data under the same conditions. In fact, no significant differences were noted between the two data. 

### 3.3. Comparison between Conventional and Established Sonotrode Extraction

Table 6 displays the comparison between the extraction of flavan-3-ols using sonotrode US at the optimal conditions established by our model and that using conventional extraction carried out according to Carciochi et al. [27].

According to the results obtained, the proposed methodology recovered 57.9% more total content of PCs than conventional extraction. Therefore, sonotrode ultrasound-assisted extraction is a more effective technique than conventional extraction for the recovery of PCs from BSGs. These data are in agreement with the data presented by Carciochi et al. [27].

Moreover, comparison with the literature is difficult because the information about the proanthocyanidin composition of BSGs is scarce. Comparing the values of proanthocyanidins obtained in this work with that obtained in barley samples, the contents of catechin, procyanidins, and prodelphynidins obtained in this work were on the same order of magnitude as those obtained in barley samples [4,8]. According to Moreira and co-workers [29], the present data also confirmed that light malt types as used for pilsner beer production contain high amounts of phenolic compounds.

In spite of proanthocyanidins being degraded at high temperatures during malting, where barley is milled, mixed with water in the mash tun, and the temperature of mash slowly increased from 37 to 78 °C to promote enzymatic hydrolysis of malt constituents [1], and during beer production, it was confirmed that a part of barley and hop proanthocyanidins still remain in the beer spent grains after beer production. Concentrations of catechin obtained at optimum sonotrode US conditions and in conventional extraction (8.96 ± 0.23 and 3.89 ± 0.36 mg∙g^−1^ d.w., respectively) were higher than that reported by Ikram et al. [30] in brewers spent grain samples (1.08 ± 0.04 µg∙100 g^−1^ d.w.). These differences could be because the catechin content of BSG varies according to barley variety, harvest time, malting and mashing conditions, and the quality and type of adjuncts added in the brewing process [1], but could also be due to the extraction method adopted for the proanthocyanidin extraction.

## 4. Conclusions

HPLC-FLD-MS was used for the determination of proanthocyanidins in brewers spent grains for the first time. A Box–Behnken experimental design was used in order to optimize the sonotrode ultrasound-assisted extraction parameters to obtain the maximum proanthocyanidin content from BSG. According to the model, the most important effect on the response came from the quadratic term of acetone/water ratio, followed by the linear term of acetone/water, the linear term of the time of extraction, and the quadratic term of US power. The highest value of proanthocyanidins was obtained at 80% acetone/water (*v/v*), 55 min, and 400 W. Finally, it was proven that sonotrode ultrasonic extraction is a more effective technique than conventional extraction method, providing a higher recovery of proanthocyanidins from BSG. 

To conclude, BSGs represent a good raw material that could be used for the extraction of bioactive compounds or could be reused for the production of functional flours. In this way, further work will be done in order to validate this hypothesis.

## Figures and Tables

**Table 1:** Box-Behnken design (BBD) with the values of the sonotrode ultrasound (US) parameters with the experimental values for the dependent response of proanthocyanins (PCs) quantified by HPLC-fluorometric detector (FLD) in brewers’ spent grain (BSG) extracts; d.w.—dry weight.

*Experiment*	*Independent Factors*			*Dependent Factor*
	X_1_	X_2_	X_3_	Total (µg∙g^−1^ d.w.)
1	50	5	240	540.04
2	100	5	240	548.25
3	50	55	240	690.90
4	100	55	240	802.25
5	50	30	80	547.91
6	100	30	80	849.32
7	50	30	400	601.43
8	100	30	400	792.07
9	75	5	80	796.40
10	75	55	80	977.69
11	75	5	400	993.15
12	75	55	400	1002.31
13	75	30	240	832.04
14	75	30	240	857.04
15	75	30	240	752.68

X1: acetone/water, X2: time, and X3: US power.

**Table 2:** Table of identification of proanthocyanidins from brewers’ spent grain extracts by HPLC-MS; Rt—retention time.

Peak	Rt (min)	Compound	[M-H]^-^
1	6.7	Catechin/epicatechin	289
2	17.6	Procyanidin dimer	577
3	19.0	Prodelphinidin dimer	593
4	21.2	Prodelphinidin dimer II	593
5	24.4	Procyanidin trimer	865
6	26.8	Prodelphinidin trimer I (monogalloylated)	881
7	29.5	Prodelphinidin trimer II (digalloylated)	897
8	32.8	Procyanidin tetramer	1153
9	33.9	Prodelphinidin tetramer (digalloylated)	1457
10	36	Procyanidin pentamer	1441
11	51.7	Polymers (degree of polymerization >5)	

**Table 3:** Table of quantification of proanthocyanidins from brewers’ spent grain extracts by HPLC-FLD expressed as µg∙g^−1^ d.w. UAE—ultrasound-assisted extraction; LOQ—limit of quantitation.

Proanthocyanidin Compounds	UAE 1	UAE 2	UAE 3	UAE 4	UAE 5	UAE 6	UAE 7	UAE 8	UAE 9	UAE 10	UAE 11	UAE 12	UAE 13	UAE 14	UAE 15
Catechin/epicatechin	8.34	9.17	10.16	9.71	8.05	10.03	10.07	10.37	9.59	10.33	10.41	8.41	9.62	9.53	8.89
Procyanidin dimer	50.08	70.49	52.50	85.90	40.06	73.45	44.02	82.34	57.47	76.36	100.92	64.17	98.56	88.97	73.94
Prodelphinidin dimer	22.68	33.01	26.09	25.96	30.44	38.86	31.93	43.95	49.16	57.03	38.74	25.68	31.04	33.97	31.60
Prodelphinidin dimer II	25.69	35.62	51.16	66.60	38.16	78.03	37.09	79.55	59.02	72.00	74.00	79.08	64.73	76.73	60.15
Procyanidin trimer	73.11	28.69	61.50	54.93	54.45	67.35	37.20	64.29	88.65	92.85	103.78	52.06	103.23	97.27	95.05
Prodelphinidin trimer I (monogalloylated)	35.58	73.86	56.78	97.85	49.08	101.98	45.60	95.27	92.53	122.39	121.94	81.68	98.98	107.81	83.69
Prodelphinidin trimer II (digalloylated)	<LOQ	48.58	<LOQ	82.52	<LOQ	80.67	<LOQ	71.26	79.53	92.77	83.62	75.12	65.34	78.03	59.54
Procyanidin tetramer	<LOQ	29.46	<LOQ	46.57	<LOQ	51.15	<LOQ	44.52	45.68	56.49	55.10	45.12	<LOQ	<LOQ	<LOQ
Prodelphinidin tetramer (digalloylated)	<LOQ	32.70	<LOQ	52.06	<LOQ	58.55	<LOQ	51.20	50.76	64.87	68.59	63.57	<LOQ	<LOQ	<LOQ
Procyanidin pentamer	<LOQ	17.64	<LOQ	26.50	<LOQ	28.01	<LOQ	19.34	24.84	35.28	30.44	42.78	<LOQ	<LOQ	<LOQ
Polymers	324.57	169.04	432.71	253.66	327.67	261.23	395.52	229.98	239.17	297.31	305.60	464.64	360.52	364.73	339.83
Total	540.04	548.25	690.90	802.25	547.91	849.32	601.43	792.07	796.40	977.69	993.15	1002.31	832.04	857.04	752.68

**Table 4:** Regression coefficients and ANOVA table.

Regression Coefficients	Total Proanthocyanidins
β_0_	−1256.27 *
Linear	
β_1_	53.07 **
β_2_	−1.19 **
β_3_	−0.68
Cross product	
β_12_	0.04
β_13_	−0.01
β_23_	−0.01
Quadratic	
β_11_	−0.33 *
β_22_	0.06
β_33_	0.00 **
*R^2^*	0.8999
*p* (regression model)	0.0074
*p* (lack-of-fit)	0.3420

* Significant at α ≤ 0.05, ** significant at α ≤ 0.1; β_1_: acetone/water ratio, β_2_: time, β_3_: US power, β_0_: regression coefficient of mean.

**Table 5:** Optimal conditions for sonotrode UAE.

Optimal Conditions	Sum of Proanthocyanidins (µg∙g^−1^ d.w.)
Acetone/ water ratio (% (*v*/*v*))	80
Time (min)	55
US power	400
Predicted (µg∙g^−1^ d.w.)	1012.7 ± 15.1
Obtained value (µg∙g^−1^ d.w.)	1023.0 ± 8.9
Significant differences between predicted and obtained value	N.S.

**N.S**.: non-significant difference.

**Table 6:** Comparison of proanthocyanidin content using sonotrode and conventional extractions (µg/g d.w.).

Proanthocyanidin Compounds	Sonotrode Extraction	Conventional Extraction
Catechin/epicatechin	8.96 ± 0.23	3.89 ± 0.36
Procyanidin dimer	66.21 ± 1.10	21.34 ± 1.04
Prodelphinidin dimer	26.08 ± 0.29	10.25 ± 0.92
Prodelphinidin dimer II	80.43 ± 1.62	39.41 ± 1.37
Procyanidin trimer	53.19 ± 1.06	18.69 ± 2.06
Prodelphinidin trimer I (monogalloylated)	83.70 ± 2.12	42.16 ± 1.89
Prodelphinidin trimer II (digalloylated)	76.14 ± 0.98	35.47 ± 1.25
Procyanidin tetramer	47.09 ± 0.63	19.36 ± 0.47
Prodelphinidin tetramer (digalloylated)	65.22 ± 1.52	20.93 ± 1.12
Procyanidin pentamer	46.81 ± 1.70	18.71 ± 0.43
Polymers	469.21 ± 6.69	200.36 ± 2.89
Total	1023.04 ± 8.9	430.57 ± 3.62

## Supplementary Materials

The following are available online at https://www.mdpi.com/2076-3921/8/8/282/s1, Figure S1: Separation of BSG proantocyanidins by HPLC-FLD.
