# Supplementary figures and images for: Optimization of Sonotrode Ultrasonic-Assisted Extraction of Proanthocyanidins from Brewers’ Spent Grains
Source: Antioxidants (Basel). 2019 Aug 6;8(8):282. doi: 10.3390/antiox8080282 (PMC6721779; doi:10.3390/antiox8080282)

Figure S1

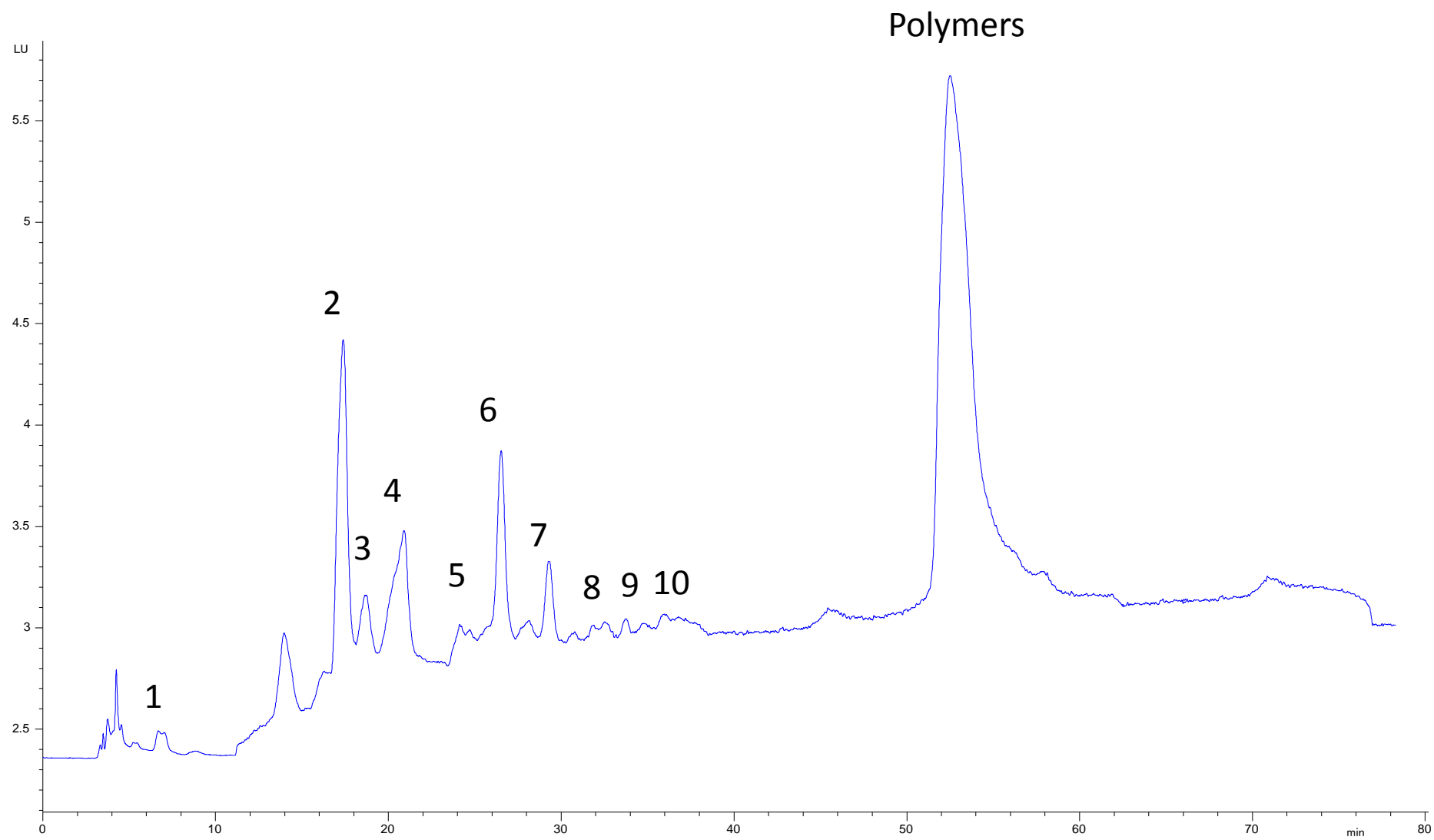

Supplement: Supplementary file 1 [file antioxidants-08-00282-s001.pdf]
